# Recollections of the Big Dallas Meeting

**Published:** 1902-06

**Authors:** 


### RECOLLECTIONS OF THE BIG DALLAS MEETING.

Some Fellows I Met.—Met Tuttle, Tuttle of the New York Polyclinic. Splendid fellow—full of fun. Very solemn looking—clerical—hair parted in the middle. The hotels were full of preachers,—big conference going on. Thought Tuttle was a preacher. Found out later that he was not a preacher by a jug-full.

Met Lanphear,—the only One and Original Great Western. Genial and delightful fellow.

Shropshire—the Sampson of Southwestern Texas. He was there, and slaughtered the Philistines—smote 'em hip and thigh. Some fellow dropped a nickel in the slot and set him to talking. He talked all the time I was there, and was talking when I left. Talking yet, I reckon; he's an eight-day-er.

West, our handsome Secretary. Popular as he is good looking. He's a last-ditcher. Scissors if I die by it. That's the sort of man I like. Glad to note that he was chosen delegate to Saratoga to represent us at meeting of the A. M. A.

Crouse—Crouse of Victoria; or rather, Crouse of Texas—there's only one Crouse. He's a scrapper, and a rattling good talker.

The Old Guard. They were all there; no use mentioning them. The Old Guard never surrenders. Big fight on over the adoption of a new constitution. At San Antonio next April something is going to happen.

Met Bangs. You all know Bangs. He's a disgusted specialist. Told me he had quit practicing medicine. Asked him why. He laughed a sickly kind of grin and said, "Its right funny. I got too lazy to keep up with the procession in general practice, and thought I'd sorter taper off, and take it easy like. Have "office hours," you know. Limited my practice to skin and hair, and advertised it as my specialty. I got more hair than I did skin. Tell you about it. Got all the bad cases of skin disease the other fellows couldn't cure,—neither could I. No money in them. The other fellows got the money before they turned them loose. But hair? Don't mention it. They came to me with superfluous hair, thin hair, hair falling out, no hair, bald all over and bald in spots, 'alopecia' the books call it. The women wanted me to turn black hair yellow, and some wanted me to turn their yellow hair back to its natural color. One woman brought me a Mexican dog, and asked if I could make the hair grow on him. Finally a young woman came and said she had a pretty little monkey that was shedding its hair, and asked me how she could make the hair stop falling off of her monkey. I quit. I'm no dog and monkey doctor. I'm now traveling for the Blue Ruin Distilling Company, Limited. See you again." And he made for the saloon across the street.

---

If at First You Don't Succeed, keep on sucking, or words to that effect. Dr. Red was elected President. Fine fellow, and will make a zealous and capable presiding officer. He has merited recognition at the hands of the Association, and the *Red Back* heartily congratulates him upon the honor, though it has been tardy, according to the doctor's way of stating the case. He said, in acknowledging the honor when he was introduced, that "continued knocking is bound to accomplish something, had been proven in his case. In 1887 his name had been mentioned for the presidency, and afterward at the Houston, San Antonio, Galveston and Waco conventions, again at the Galveston convention in 1901, and finally at this convention. It was, he said, an honor to be President of the State Medical Association, particularly so after having been twice honored with the position of First Vice-President."

---

Dr. R. E. Nicholson moved that the beautiful and accomplished daughter of the retiring President, who had graced this meeting with her presence, be elected honorary daughter of the Association. The motion was greeted with cheers, and was unanimously carried. Miss Hudson being absent at the time, her father, Dr. Hudson, acknowledged the compliment.

---

A Study in Black and White.—Dallas chuck full of people. Big Methodist Conference in session. Five hundred priests, prelates, preachers and bishops, all in black and white; solemn and serene. Three hundred doctors; medical convention. Hotels crowded. The Oriental putting cots in the corridors. Our handsome and dignified State Health Officer, George R. Tabor, came in late. Immaculate black suit, white tie, and a broad expanse of white vest spread over an aldermanic bay-window. Dignified, clean-shaved, bald and bland, he approached the hotel clerk and asked for a room. The clerk sized him up, and asked: "Are you a member of the Conference?" "Am I a member of the *Conference?*" said the doctor, transfixing him with his eagle eye. "I'm the Bishop of the Austin Beat." "Front," said the clerk. "Show the Bishop to No. One, next to the ladies' parlor." And the band in the balcony discoursed a harmony of sweet sounds.

TAYLOR HUDSON made a good presiding officer. Got a little rattled when the report of the Committee on Revision of Constitution was up. He allowed it to be side tracked and the whole question to be reopened, Shropshire in the lead. It got into the circumlocution office, and we had an edifying object lesson in how not to do it. The whole thing was put off a year, and the fight will be reopened at San Antonio next April. It is shaping into a sectional fight over the already organized district societies, the North Texas being the largest, and it will require skillful steering between Scylla and Charybdis to avoid a rupture. *Selah.*

---

THE GOVERNOR favored the Association with a speech. It was most gratifiying to hear him declare his great interest in the public health, and favor most earnestly a State Board of Health.

---

TWO PATRIARCHS OF THE PROFESSION, two relics of the Army Medical Corps of the Confederacy, Dr. Samuel Hollingsworth Stout, Medical Director of all the Confederate Hospitals, aged 80, and Dr. Thomas C. Osborn, of Cleburne, the originator of the bichloride treatment of smallpox, aged 85, were presented to the Association by Dr. Daniel, and they received an ovation which was very gratifying to them and their friends.

---

THE BRAZOS VALLEY MEDICAL SOCIETY held its regular semi-annual meeting in the beautiful little city of Marlin, May 13th and 14th ult. There was a fairly good attendance, and the meeting was a delightful reunion, much enjoyed by ye senior of ye *Red Back.* It is much to be regretted that our old war veteran Briggs, the long-time secretary and the wheelhorse of the bandwagon, was sick (sic?) and unable to attend. Marlin should become—and will become when it is better advertised and more widely known—a favorite resort, not only for chronic invalids who seek its hot and cold mineral waters and baths, but for pleasure seekers who want cool and comfort during our long summers. The new Arlington is a vision of beauty—and a joy forever. It was a revelation to us. I had no idea there was such a magnificent and beautiful hotel in the South, and the *cuisine* and general management under the courteous and genial host, Col. Harrison, are above criticism. It would be a luxury to even be sick at such a delightful place. Col. Harrison furnished the spread (the B.-V. always has a spread), at the New Arlington, and such a banquet has not been set before a lot of hungry doctors, made dry by much—if not good—talking, in many a day. The tables were beautiful; the flowers beautiful; but the fairest and most beautiful of all were the ladies of Marlin who graced the festive board with their presence and inspired the toast responders with their smiles and bright eyes, Bright shone the lamps over fair women and brave doctors; and when the music 'rose with its voluptuous swell, soft eyes looked love to eyes which spake again, and all went as merry as a marriage bell, or something like that.

